# Lung Immune Prognostic Index Could Predict Metastasis in Patients With Osteosarcoma

**DOI:** 10.3389/fsurg.2022.923427

**Published:** 2022-07-08

**Authors:** Xuanhong He, Yitian Wang, Qiang Ye, Yang Wang, Li Min, Yi Luo, Yong Zhou, Chongqi Tu

**Affiliations:** Department of Orthopedics, Orthopedic Research Institute, West China Hospital, Sichuan University, Chengdu, China

**Keywords:** lung immune prognostic index (LIPI), osteosarcoma, metastasis, lactate dehydrogenase (LDH), derived neutrophil to lymphocyte ratio (dNLR)

## Abstract

**Background:**

The lung immune prognostic index (LIPI), composed of serum lactate dehydrogenase (LDH) and the derived neutrophil to lymphocyte ratio (dNLR), is a novel prognostic factor of lung cancer. The prognostic effect of the LIPI has never been verified in osteosarcoma.

**Methods:**

We retrospectively reviewed the osteosarcoma patients with metachronous metastasis from January 2016 to January 2021 in West China Hospital. We collected and analyzed the clinical data and constructed the LIPI for osteosarcoma. The correlation between the LIPI and metastasis was analyzed according to the Kaplan–Meier method and Cox regression analysis with hazard ratios (HRs) and 95% confidence intervals (CIs). Univariate analysis and multivariate analysis were conducted to clarify the independent risk factors of metastasis. The nomogram model was established by R software, version 4.1.0.

**Results:**

The area under the curve (AUC) and best cutoff value were 0.535 and 91, 0.519, and 5.02, 0.594 and 2.77, 0.569 and 227.14, 0.59 and 158, and 0.607 and 2.05 for ALP, LMR, NLR, PLR, LDH, and dNLR, respectively. The LIPI was composed of LDH and dNLR and showed a larger AUC than other hematological factors in the time-dependent operator curve (t-ROC). In total, 184 patients, 42 (22.8%), 96 (52.2%), and 46 (25.0%) patients had LIPIs of good, moderate, and poor, respectively (*P* < 0.0001). Univariate analysis revealed that pathological fracture, the initial CT report of suspicious nodule, and the NLR, PLR, ALP, and the LIPI were significantly associated with metastasis, and multivariate analysis showed that the initial CT report of suspicious nodule and the PLR, ALP, and LIPI were dependent risk factors for metastasis. Metastatic predictive factors were selected and incorporated into the nomogram construction, including the LIPI, ALP, PLR, initial CT report, and pathological fracture. The C-index of our model was 0.71. According to the calibration plot, this predictive nomogram could accurately predict 3- and 5-year metachronous metastasis. Based on the result of decision curve and clinical impact curve, this predictive nomogram could also help patients obtain significant net benefits.

**Conclusion:**

We first demonstrated the metastatic predictive effect of the LIPI on osteosarcoma. This LIPI-based model is useful for clinicians to predict metastasis in osteosarcoma patients and could help conduct timely intervention and facilitate personalized management of osteosarcoma patients.

## Introduction

Osteosarcoma is the most prevalent primary malignant bone tumor and mainly involves children, adolescents, and the elderly (1, 2). With the combination of neoadjuvant chemotherapy and surgical excision, the 5-year survival rate of osteosarcoma patients without metastasis ranged from 60% to 70% (3, 4). However, patients with metastasis have a poor prognosis with a dismal survival rate of approximately 20% (5, 6). Almost all patients had subclinical micrometastatic lesions at diagnosis, but metastasis could be successfully detected in only 20% of patients at their first visit to the hospital (7, 8). In addition, approximately 25%–35% of patients without initial metastasis develop distant metastasis during the clinical course (9, 10). Notably, patients with metastasis were more likely to become insensitive to chemotherapy (11). Although huge efforts have been invested to develop novel drugs, such as immune checkpoint and molecular targeted inhibitors, limited progression has been achieved, and the outcome of metastatic patients is still unsatisfactory (12). Therefore, diagnosing metastasis in the early stage and detecting micrometastasis are crucial to promote the long-term survival rate (12). However, there is still no valid method to evaluate the metastasis status and predict the probability of metastasis in osteosarcoma patients.

Inflammation is associated with the growth, progression, and metastasis of cancer, including osteosarcoma (13). Several factors were reported to be correlated with the overall survival of osteosarcoma patients, including alkaline phosphatase (ALP), lymphocyte–monocyte ratio (LMR), neutrophil–lymphocyte ratio (NLR), platelet–lymphocyte ratio (PLR), LDH, and dNLR. However, the predictive effect of inflammatory factors on metastasis has seldom been explored in osteosarcoma patients. The lung immune prognostic index (LIPI), combining LDH and the dNLR, was first introduced by Mezquita et al in metastatic non-small-cell lung cancer (NSCLC), in which pretreatment LIPI was associated with poor outcome for immune checkpoint inhibitors (14). Other studies also demonstrated the prognostic ability of the LIPI in extrapulmonary cancer (15–17). Nonetheless, the prognostic effect of the LIPI has not been explored in osteosarcoma.

Therefore, in this study, we investigated the prediction ability of the LIPI in metastasis and constructed a predictive model to evaluate the probability of distant metastasis.

## Patients and Methods

### Patients

From January 2016 to January 2021, all osteosarcoma patients in the Musculoskeletal Tumor Center of West China Hospital were reviewed. The inclusion criteria were as follows: (1) patients with high-grade osteosarcoma diagnosed by histopathology; (2) patients with complete hematological test results before neoadjuvant chemotherapy; (3) patients who received standard treatment at West China Hospital; and (4) patients who developed distant metastasis during the follow-up. The exclusion criteria were as follows: (1) patients who had received neoadjuvant chemotherapy before their first consultancy in our hospital; (2) patients with hematological diseases; (3) patients with other malignancies; and (4) patients who had not received standard treatment (patients who were misdiagnosed and mistreated or failed to complete postoperative chemotherapy). (5) patients with distant metastasis at the first diagnosis. Eventually, 184 patients were included in this study, and each of them was followed up regularly until death or January 2022. During the follow-up, patients were advised to be reexamined every 3 months in the first year postoperatively, every 4 months in the second year postoperatively, every 5 months in the third year postoperatively, every 6 months in the fourth and fifth years postoperatively, and every year more than 5 years postoperatively. This study was approved by the Ethics Committee of West China Hospital, and written informed consent was obtained from all participants**.**

### Data Collection and Analysis

Before neoadjuvant chemotherapy, hematological factors, including leukocyte count (Leut#), neutrophil count (Neut#), lymphocyte count (LYMPH#), monocyte count (MONO#), platelet count (PLT), lactate dehydrogenase (LDH), and alkaline phosphatase (ALP), were extracted from the first blood routine of 184 patients before neoadjuvant chemotherapy. The formulas for calculating the NLR, PLR, LMR, and dNLR are as follows: NLR = Neut#/LYMPH#, PLR = PLT/LYMPH#, LMR = LYMPH#/MONO#, and dNLR = Neut#/(Leut#-Neut#). In addition, age, sex, tumor site, pathologic fracture status, initial chest CT report (with suspicious nodules on chest CT), and tumor metastasis status were collected from the patients' medical records. The initial chest CT refers to the patient's most recent pretreatment chest CT. According to a previous study, suspicious pulmonary nodules were defined as non-calcified nodules with a maximum diameter of <10 mm (18). Metastasis-free survival (MFS) was the end point of this study and was calculated from the date of diagnosis to the date of metastasis or last follow-up. In the overall cohort, the optimal cutoff value for each hematological marker was calculated based on the time-dependent receiver operating curve (ROC) and converted into a binary variable according to the cutoff value.

### Establishment and Validation of the LIPI

The LIPI in osteosarcoma was established by combining LDH and dNLR. Then, the metastasis prediction effect of the LIPI was compared with that of other hematological factors, and clinical characteristics by time-dependent ROC metastasis probability between different groups of patients were analyzed. To verify whether the LIPI is an independent predictive factor of osteosarcoma patients with metastasis, we conducted univariate and multivariate analyses. Significant factors in univariate analyses were then subjected to multivariate analyses to determine independent prognostic factors.

### Construction and Evaluation of the Metastasis Prediction Nomogram

After a multistep screening process, metastasis prediction factors were selected and incorporated into the nomogram construction. For each patient, the total point was equal to the sum of the points of all factors. The relationships between the total points and the probability of MFS are shown at the bottom of the nomogram. The discrimination ability and accuracy of the nomograms were evaluated by Harrell's concordance index and calibration curve, respectively. The diagonal acts as a reference line and represents the best prediction. Decision curve analysis (DCA) was used to evaluate the clinical application of the nomogram by estimating the net benefits at different threshold probabilities. The clinical impact curve was also drawn to predict reduction intervention probability per 100 patients.

### Statistical Analysis

The Kolmogorov–Smirnov test was used to assess whether continuous variables were normally distributed, and the Mann–Whitney U test or Spearman correlation analysis was used to assess differences between continuous variables according to the results. Categorical variables were evaluated using the chi-square test and Fisher's exact test based on the number of individuals in each group. All statistical analyses were conducted using R software, version 4.1.0 (Institute for Statistics and Mathematics, Vienna, Austria). *P* values < 0.05 were considered to indicate statistical significance.

## Results

### Patient Demographics and Optimal Cutoff Values of Hematological Factors

Patient demographics are shown in Table 1. A total of 184 patients were enrolled in this study, of which 107 were males and 77 were females. The age of the patients ranged from 7 to 67 years, with a mean age of 21 years. A total of 176 tumors mainly located at the extremities (96.0%), and only 8 tumors (4.0%) located at the extra-extremities. Pathological fracture at diagnosis was found in 19 (11.2%) patients, and metastasis at diagnosis was found in 64 (34.8%) patients. The mean time to metastasis was 14.7 months after diagnosis.

**Table 1:** Patients demographics.

	Patients	LIPI			*P*-value
		Poor	Moderate	Good	
Total patients	184	46	96	42	—
Age (years)					
>21	128	30	74	24	0.049
≤21	56	16	22	18	
Gender					
Male	107	28	52	27	0.493
Female	77	18	44	15	
Tumor location					0.014
Extremities	176	41	95	40	
None extremities	8	5	1	2	
Pathological fracture					
Yes	19	8	8	3	0.207
No	165	38	88	39	
Metachronous Metastasis					0.000
Yes	64	26	32	6	
No	120	20	64	36	
Initial CT report					0.599
No-abnormalities	117	27	61	29	
Pulmonary. nodules	67	19	35	13	
ALP (IU/l)					0.020
>91	91	29	48	14	
≤91	93	17	48	28	
LMR					0.759
>5.02	58	6	33	19	
≤5.02	126	40	63	23	
NLR					0.000
>2.77	73	44	27	2	
≤2.77	111	2	69	40	
PLR					0.019
>227.14	42	16	22	4	
≤227.14	142	30	74	38	
LDH (IU/l)					0.201
>158	120	46	74	0	
≤158	64	0	22	42	

*LIPI, lung immune prognostic index; Initial CT report, initial computed tomography report; ALP, alkaline phosphatase; LMR, lymphocyte–monocyte ratio; NLR, neutrophil–lymphocyte ratio; PLR, platelet–lymphocyte ratio; LDH, lactate dehydrogenase.*

**Figure 1:**
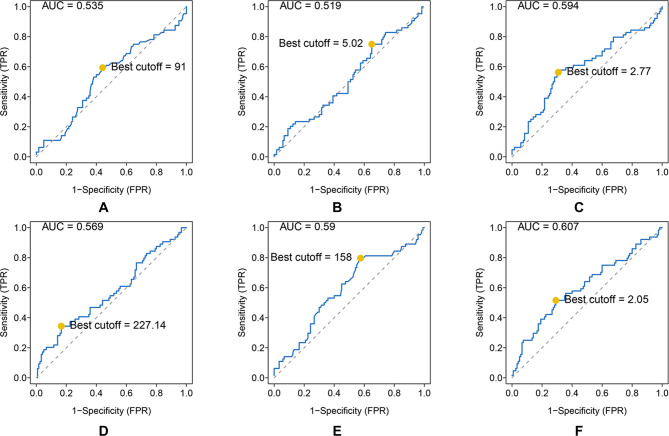
ROC analysis of different hematological biomarkers. (**A**–**F**) The AUC and best cutoff values of ALP, LMR, NLR, PLR, LDH, and dNLR are shown, respectively. The vertical axis represents the sensitivity and the horizontal axis represents the 1-specificity.

As shown, the AUC and best cutoff values were 0.535 and 91, 0.519 and 5.02, 0.594 and 2.77, 0.569 and 227.14, 0.59 and 158, and 0.607 and 2.05 for ALP, LMR, NLR, PLR, LDH, and dNLR, respectively (Figures 1A–F).

### Establishment and Validation of the LIPI in Osteosarcoma

**Figure 2:**
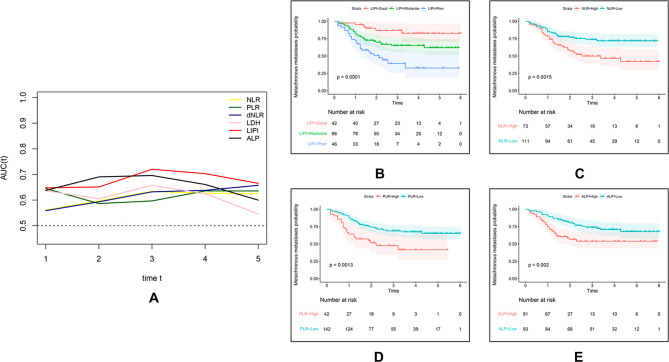
Comparison of different hematological factors in predicting the metastatic probability. (**A**) The difference of predictive ability is shown in a time-dependent ROC curve, in which a larger AUC value means a better metastatic predictive ability. (**B**,**C**) Metastatic predictive ability of different hematological factors in 184 osteosarcoma patients.

On the basis of the results of Figure 1, we constructed the LIPI by combining the LDH and dNLR. According to the t-ROC curve, the LIPI showed a better metastasis predictive ability with a larger AUC than other predictive biomarkers, including the NLR, PLR, LDH, dNLR, and ALP (Figure 2A). The LIPI graded osteosarcoma patients into three groups according to the abnormal values of LDH and dNLR. In a total of 184 patients, 42 patients were good (22.82%), 96 patients were moderate (52.17%), and 46 patients were poor (25.00%). Patients with poor LIPI were more likely to develop distant disease than patients in the other two groups (*P* < 0.0001). Other hematological biomarkers were also associated with the metastasis prediction of osteosarcoma. Patients were divided into two groups based on a comparison with the optimal cutoff value. A total of 73 patients showed a high (39.67%) NLR and 111 patients had a low (60.32%) NLR, and patients with a high NLR were more likely to develop distant metastasis (*P* = 0.0015). A total of 42 patients showed a high (22.82%) PLR, and 142 patients had a low (77.17%) PLR, and patients with a high PLR had a higher probability of developing distant metastasis (*P* = 0.0013). A total of 91 patients showed a high (49.46%) ALP, and 93 patients had a low (50.54%) ALP, and patients with a high ALP were more inclined to develop distant metastasis (*P* = 0.002).

### Univariate Analysis and Multivariate Analysis

**Figure 3:**
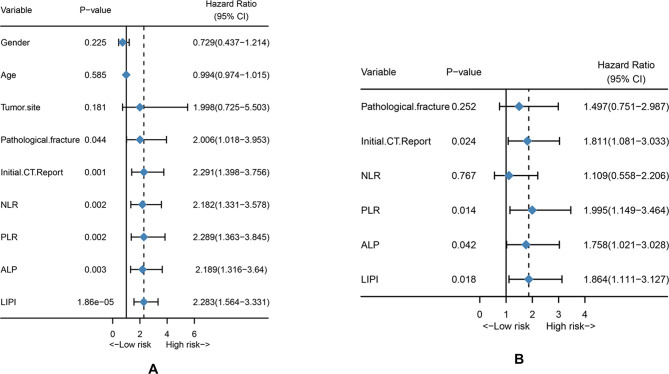
Univariate analysis and multivariate analysis. (**A**) Univariate analysis of clinical features and hematological factors. (**B**) Multivariate analysis of significant clinical factors and hematological factors.

The univariate and multivariate Cox analysis for MFS is shown in Table 2. Univariate analysis revealed that pathological fracture (HR = 2.006 (1.081–3.953), *P* = 0.044), initial CT report (HR = 2.291 (1.398–3.756), *P* = 0.001, NLR (HR = 2.182 (1.331–3.578), *P* = 0.002), PLR (HR = 2.289 (1.363–3.845), *P* = 0.002), ALP (HR = 2.189 (1.316–3.640), *P* = 0.003), and LIPI (HR = 2.283 (1.564–3.331), *P* = 1.86e^−05^) were all significantly associated with osteosarcoma metastasis (Figure 3A). Then, the significant values were subjected to multivariate analyses to determine independent prognostic factors. The results demonstrated that the initial CT report (HR = 1.811 (1.081–3.033), *P* = 0.024), PLR (HR = 1.995 (1.149–3.464), *P* = 0.014), ALP (HR = 1.758 (1.021–3.028), *P* = 0.042), and LIPI (HR = 1.864 (1.111–3.127), *P* = 0.018) were all independent metastasis prediction factors in patients with osteosarcoma (Figure 3B).

**Table 2:** Univariate and multivariate Cox analysis for MFS.

	Univariate analysis		Multivariate analysis	
	HR (95% CI)	*P*-value	HR (95% CI)	*P*-value
Gender	0.729 (0.437–1.214)	0.225		
Age	0.994 (0.974–1.015)	0.994		
Tumor site	1.998 (0.725–5.503)	0.181		
Pathological fracture	2.006 (1.018–3.953)	0.044	1.497 (0.751–2.987)	0.252
Initial CT report	2.291 (1.398–3.756)	0.001	1.811 (1.081–3.033)	0.024
NLR	2.182 (1.331–3.578)	0.002	1.109 (0.558–2.206)	0.767
PLR	2.289 (1.363–3.845)	0.002	1.995 (1.149–3.464)	0.014
ALP	2.189 (1.316–3.64)	0.003	1.758 (1.021–3.028)	0.042
LIPI	2.283 (1.564–3.331)	1.86e-05	1.864 (1.111–3.127)	0.018

*MFS, metastasis-free survival; Initial CT report, initial computed tomography report; NLR, neutrophil–lymphocyte ratio; PLR, platelet–lymphocyte ratio; ALP, alkaline phosphatase; LIPI, lung immune prognostic index.*

### Construction and Validation of LIPI-Based Nomogram

**Figure 4:**
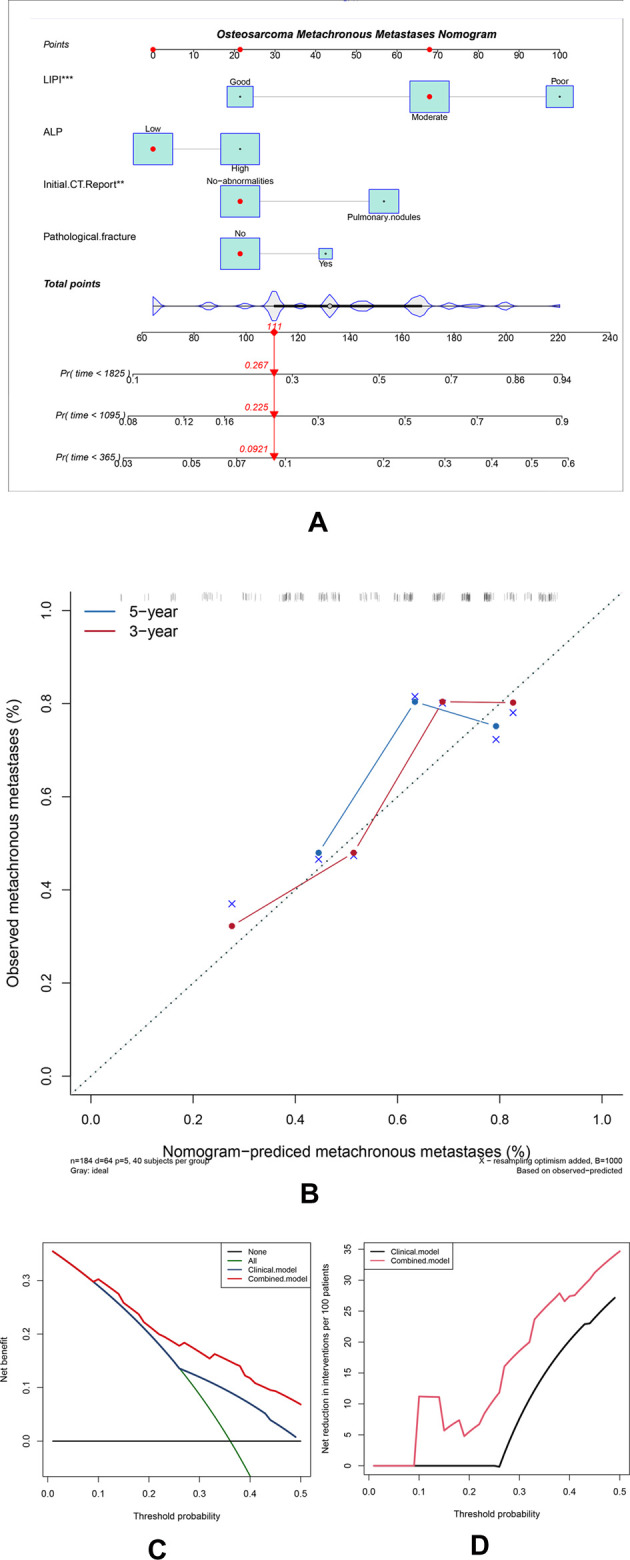
Construction and validation of the osteosarcoma metachronous metastases nomogram. (**A**) The nomogram was constructed by combining the LIPI, ALP, initial CT report, and pathological fracture, and the sum of the scores for each covariate was the nomogram total score. (**B**–**D**) This nomogram was validated by the calibration curve, decision curve analysis, and clinical impact curve.

To investigate the clinical practice of the LIPI, we also developed a nomogram combining the LIPI with clinical characteristics in patients with osteosarcoma. The hematological factors (LIPI and ALP) and clinical characteristics (initial CT report and pathological fracture) were included in this nomogram to predict the 1-, 3-, and 5-year metastasis probability for osteosarcoma patients. It is worth noting that the variables of the LIPI and PLR contained the index of lymphocyte. Therefore, the weight of the lymphocyte, which was evaluated two times, may influence the accuracy of the currently established nomogram. Thus, we excluded the PLR from the construction of this nomogram. As shown, Cox proportional hazards regression assigned a score based on the hazard ratio for each covariate, and the sum of the scores for each covariate was the nomogram total score (Figure 4A). According to the calibration curve, the 3-year and 5-year predicted metachronous metastases curves were consistent with the diagonal line in the calibration curve, which meant that this nomogram could accurately predict 3-year and 5-year metachronous metastases. The C-index of this nomogram was 0.71 (Figure 4B). The clinical benefits of nomograms through DCA and clinical impact curves were also explored. According to Figures 4C, D, the combined model tended to help patients obtain significant net benefits compared with the clinical model (Figures 4C, D).

### Comparison of the Metastasis Prediction Ability of the LIPI with Clinical Features

**Figure 5:**
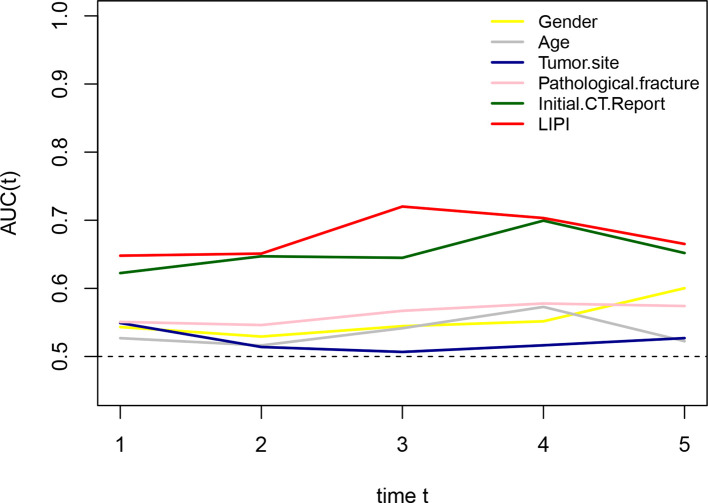
Comparison of the metastatic predictive effect between LIPI and clinical features. A larger AUC in the t-ROC means a better predictive ability.

To compare the metastasis prediction ability of the LIPI with clinical features, including sex, age, tumor site, pathological fracture, and the initial CT report, we plotted time-dependent ROC curves. As shown in Figure 5, the LIPI had a larger AUC, and its metastasis prediction ability was significantly higher than that of the clinical features.

## Discussion

Osteosarcoma is the most common malignant bone tumor and predominantly affects children and the elderly (1, 2). Metastasis is a well-known poor prognostic factor, and the long-term survival rate of patients with metastasis is only 20%, which is much lower than the 60%–70% of patients without metastasis (12). Patients with metachronous metastasis was the main cause of the dismal of treatment failure (19, 20). Although much effort has been made to explore novel drugs, including immune checkpoint and molecular targeted inhibitors, limited progress has been achieved (20, 21). Subclinical metastasis was believed in almost all osteosarcoma patients, while a small proportion of them could be detected during the first diagnosis (12). Therefore, establishing a metastasis predictive model that could evaluate the metastasis status and predict the metastasis probability is necessary.

Cancer-related inflammation is recognized as a novel hallmark of cancer and contributes to the development of cancer by stimulating proliferation, angiogenesis, and metastasis, reducing the response to chemotherapy agents and subvert adaptive immunity (22, 23). The association between osteosarcoma and inflammation was also extensively explored. Several hematological factors were reported to be valid in predicting the outcome of osteosarcoma patients, including LDH, the NLR, and so on (24–27). However, the association between hematological inflammatory factors and osteosarcoma metastasis has seldom been explored. According to Araki et al., the PLR and neutrophil count were reported to be independent risk factors for metastasis in osteosarcoma patients, but this hematological index was single and not persuasive (28). Therefore, identifying a comprehensive inflammatory indicator that can reflect the inflammatory status *in vivo* may be the potential direction. The LIPI, composed of LDH and the dNLR, is an integrated inflammation index relevant to the prognosis of lung and extra-lung cancer (14). The LIPI could help make treatment decisions in special cancers, such as the application of immune checkpoint inhibitors, chemotherapy, and epidermal growth factor receptor tyrosine kinase inhibitors in NSCLC (14, 29). Nevertheless, the prognostic effect of the LIPI in osteosarcoma has never been reported before. In this study, we first explored the association between the LIPI and osteosarcoma and constructed a LIPI model in osteosarcoma. This LIPI graded patients into three groups: good, moderate, and poor (Figure 2B). Compared with other hematological factors, the LIPI distinguished patients at different metastasis risk levels and was more accurate in predicting the probability of metastasis according to the t-ROC (Figure 2A). Simultaneously, univariate analysis and multivariate analysis showed that the LIPI, accompanied by the initial CT report, PLR, and ALP, was a significant independent predictive factor for metastasis (Figures 3A, B), implying that the inflammatory state *in vivo* was correlated with the progression and metastasis of osteosarcoma patients. Compared with other single metastatic predictors such as the PLR, LIPI can evenly divide patients into three layers according to the metastatic risk degree, which helps to differentiate patients at a high risk of metastases from patients with a low PLR. This more accurate predictive model may facilitate the development of personalized treatment decision, as well as avoid unnecessary intervention.

A nomogram is a precise and convenient mathematical model that can predict the specific end point and a useful tool to quantify and evaluate risks, which can help make beneficial clinical decisions for patients (30). Furthermore, we integrated inflammatory factors (including the LIPI and ALP) and clinical features (initial CT report, pathological fracture) to construct the LIPI-based metastasis predictive nomogram (Figure 4A). Each of these factors has its corresponding score. For each osteosarcoma patient, the sum of the scores was equal to the sum of all metastasis predictors, and each sum had corresponding rates of 1-year, 3-year, and 5-year MFS. For example, an osteosarcoma patient who was classified as having a moderate LIPI, a low ALP, no-abnormalities in initial CT reports, and no pathological fracture pretreatment, had a total score of 145 points, corresponding to the metastatic rates of 7.39%, 18.8%, and 22.9% in 1-year, 3-year, and 5-year MFS, respectively. Therefore, this patient had a high 3-year and 5-year risk of metastasis, and a closer follow-up from the second year is recommended to timely detect and manage metastases. According to the validation results, this predictive model could accurately predict the 3-year and 5-year metastasis rates (Figures 4B–D). These results were inspiring, because precisely evaluating the probability of metastasis has great clinical significance, which can assist clinicians in timely diagnosing and conducting appropriate intervention to patients with potential metastasis, ultimately improving the prognosis.

Some attempts have been made to establish a metastasis predictive model; however, most of them merely focused on a single inflammatory factor or clinical feature, which is difficult to represent the disease state of those patients (28, 31–33). In this study, we also verified that the LIPI had an advantage over predicting the metastasis status over other single inflammation factors and clinical features (Figures 2A, 5). In addition, we also compared the combined model (the LIPI with other prognostic factors) with the clinical model, and the results revealed that this combined model had a better predictive ability. Therefore, we believe that this combined model based on the LIPI could to some extent be more representative of the disease state in osteosarcoma patients with potential metastasis and could further predict the probability of distant metastasis. In addition, compared with the current micrometastasis detection methods, such as immunomagnetic detection assays and molecular imaging with specific molecular probes, our LIPI-based predictive model is simpler, easier to implement, and economical (12). Of course, the clinical practicability of this model requires further investigation.

Notably, during the construction process of this predictive model, we integrated the initial CT report into our model, which has seldom been reported before. Pulmonary metastasis is the most frequent site of metastasis, accounting for 85%–90% of metastatic patients (20). Most of the nodules on breast CT presented as indeterminate nodules and were hard to distinguish from metastatic lesions, which always confuses clinicians and patients, and induces uncertainty in treatment options (34). Herein, we reviewed the initial breast CT reports of 184 patients and conducted a correlation analysis between indeterminate nodules and metastatic lesions. Our results demonstrated that the initial CT report was an independent factor of metastasis (Figures 3A, B). Therefore, for osteosarcoma with indeterminate nodules on CT reports at first diagnosis, we recommended more frequent chest high-resolution CT and positive intervention, which was consistent with previous studies (34, 35).

The LIPI was combined with LDH and the dNLR in this study. LDH is a crucial enzyme in the metabolism of lactate and is considered to contribute to the initiation and development of cancer by accelerating tumor metastasis and angiogenesis (36). LDH was reported to be associated with poor prognosis in osteosarcoma patients (37). In cancers, including carcinoma, lung cancer, and colorectal cancer, higher serum levels were associated with metastatic tendency and were considered a useful metastatic biomarker (38–41). The dNLR is also an important inflammatory biomarker that can be used to measure the inflammatory state in cancers (14). Given its simplicity and convenience, the dNLR has been widely applied in predicting the outcome of different cancers, and a higher dNLR is associated with poor prognosis in patients with different cancers (42–45). However, the predictive effect of the dNLR in osteosarcoma remains unclear. We first explored its function in osteosarcoma when combined with LDH. According to our findings, we believe that both LDH and the dNLR are meaningful in predicting metastasis in osteosarcoma patients.

We must acknowledge that there are several limitations in this study. First, this single-center study may have resulted in selection bias. Second, this study is a retrospective study, and, thus, there may be a bias in the collection and interpretation of the patients and material. Despite these drawbacks, this is the first study to explore the clinical significance of the LIPI in osteosarcoma, which lays the foundation for further studies of the LIPI in osteosarcoma, such as the response of osteosarcoma to immunotherapy. Moreover, in our future study, we plan to include more cases and more metastatic predictive factors to improve the LIPI-based nomogram, ultimately increasing its clinical practicality.

## Conclusion

We first demonstrated the metastatic predictive effect of the LIPI in osteosarcoma. This LIPI-based model is useful for clinicians to predict metastasis in osteosarcoma patients and could help conduct timely intervention and facilitate personalized management of osteosarcoma patients.

## Data Availability

The original contributions presented in the study are included in the article/**Supplementary Material**, and further inquiries can be directed to the corresponding author/s.
